# Cancer and COVID-19: economic impact on households in Southeast Asia

**DOI:** 10.3332/ecancer.2020.1134

**Published:** 2020-11-03

**Authors:** Yek-Ching Kong, Veni-Venusha Sakti, Richard Sullivan, Nirmala Bhoo-Pathy

**Affiliations:** 1 Centre for Epidemiology and Evidence-Based Practice, Faculty of Medicine, University of Malaya, 50603, Lembah Pantai, Kuala Lumpur, Malaysia; 2 King’s College London, Institute for Cancer Policy, Guy’s Hospital, Great Maze Pond, London SE1 9RT, United Kingdom

**Keywords:** cancer, COVID-19, financial toxicity, Southeast Asia

## Abstract

The ongoing COVID-19 pandemic may worsen the existing financial vulnerabilities of cancer survivors who may be experiencing a double financial hit, both from cancer-induced financial toxicity as well as economic strains arising from loss of income and prolonged unemployment following the pandemic. The impact of the pandemic is likely to be more pronounced on cancer survivors living in resource-limited settings, such as in Southeast Asia. As health care systems in the region try to streamline resources and accommodate the influx of patients from COVID-19, many in the cancer community have experienced severe disruptions in their care. The delays and disruption of timely access to cancer care could lead to patients presenting with worsened conditions and at more advanced cancer stages in which treatment options tended to be costlier. Similar to countries around the world, the various forms of movement restrictions that were enforced have aggravated the rates of unemployment, loss of wages and the limited access to support from family or friends around Southeast Asia. The economic impact of COVID-19 hits even harder on the large proportion of the population in the region that works in the informal sector, who are often one paycheque or one episode of illness away from financial catastrophe. More worryingly, the lack of a robust social security system in many Southeast Asian countries, especially in terms of income protection, could ultimately force many cancer survivors to choose between paying for their treatments, or to forego treatments, and feed their families. Early identification of cancer patients experiencing financial toxicity following the pandemic will enable timely and appropriate interventions to be undertaken by various stakeholders, potentially averting a cascade of other economic fallouts that may last for years after cancer treatment.

## Introduction

Financial toxicity is a term that was coined to describe the harmful financial burden experienced by people with cancer, encompassing both the objective financial burden and subjective financial distress [1–3]. The financial distress brought about by a cancer diagnosis may not only have a negative impact on patients’ treatment choice, and adherence to treatment, but also lead to deterioration in the quality of life and psychological well-being of patients and their families [4–6]. In the effort to cope with the financial strains, many cancer patients have reported remedying rising costs by limiting medication use and skipping clinic appointments [7]. Moreover, patients experiencing cancer-related financial strain have been associated with a substantially higher risk of depression [8] and were also more likely to report poor satisfaction with social activities and relationships [9]. Compared to those without cancer, cancer patients were also more likely to be unemployed [10], or file for bankruptcy [11].

The ongoing COVID-19 pandemic may worsen the existing financial vulnerabilities of cancer survivors. This may be especially true given that people with cancer may now take on a double financial hit, both from cancer-induced financial toxicity as well as economic strains arising from loss of income and prolonged unemployment following the pandemic. More worryingly, the impact of the pandemic is likely to be more pronounced on cancer survivors living in resource-limited settings, such as in Southeast Asia, who may end up enduring even more financial sufferings compared to their counterparts living in affluent settings.

## Economic impact of cancer and COVID-19 in Southeast Asia

More than 2 million cases of cancer are diagnosed annually [12], with an estimated 5-year prevalence of 4 million people living with cancer in Southeast Asia, where the healthcare systems and financing mechanisms are varied and diverse [13]. Through the ASEAN CosTs In ONcology (ACTION) study, comprising close to 10,000 cancer patients from eight low- and middle-income countries in Southeast Asia, we had previously shown that close to 50% of cancer patients in the region experienced catastrophic health payments (out-of-pocket payments exceeding 30% of annual household income) just within a year after diagnosis [14], leading in many instances to economic hardship and impoverishment [15]. Country-specific analyses revealed similar findings [16–18]. Our recent qualitative inquiry in a multiethnic population of Malaysian women with breast cancer had also revealed that unmet financial needs related to cancer treatment and healthcare might exist even among patients from high-income groups, as well as those with health insurance [19]. These needs range from financial assistance to cover out-of-pocket payments for medical and non-medical costs following cancer diagnosis, to navigation through the complex system to claim medical insurance or social security benefits, and return to work assistance.

In the effort to curb the spread of COVID-19, various forms of movement restrictions, including the closing of national borders, have been implemented in many Southeast Asian countries. In Malaysia, the Movement Control Order, enforced on 18 March 2020, brought about the closure of all non-essential services including learning institutions, a ban on interstate travel, limited business operating hours as well as 10 km radius travel restriction for purchase of essentials [15]. The Movement Control Order was only relaxed on 10 June 2020, marking the start of the recovery phase of the movement control. In neighbouring Singapore, the Circuit Breaker was initiated on 1 April 2020 and initially slated to last for about a month, till 4 May 2020 [21]. Nonetheless, the surging counts in daily new cases prompted the government to extend the Circuit Breaker to 1 June 2020, with even tighter restrictions. In Thailand, the state of emergency which started on 25 March 2020, enforcing night curfews and restrictions on inter-provincial and border travels, had recently been extended until the end of September [22, 23]. Despite having the longest and strictest lockdown in the world from 15 March to 1 June 2020, the Philippines, which has recently overtaken Indonesia with the most COVID-19 cases in Southeast Asia, went back into lockdown on 4 August 2020 [24, 25].

With the movement restrictions in place and suspension of public facilities due to the COVID-19 pandemic, accessibility to hospitals and treatment may be a challenge for the underprivileged patients, including those from rural areas, and low-income groups [26]. Health care systems are also facing interruptions in their services as they try to accommodate the influx of patients from COVID-19. For instance, during the movement control order in Malaysia, many nonurgent treatments, elective surgeries, hospital admissions and follow-up appointments were either delayed or cancelled in order to streamline resources for the COVID-19 pandemic. Subsequently, the oncology departments in many major public hospitals in Malaysia, which are mostly COVID-designated facilities, scaled down elective surgeries to two days a week, shortened the clinic hours, prioritized radiotherapy and chemotherapy administrations based on the magnitude of potential treatment benefits and also postponed imaging procedures to monitor tumour growth [27]. This has severely disrupted cancer diagnosis, active treatments and routine follow-ups of many in the cancer community. The delays and disruption of timely access to cancer care bring about severe implications as it could lead to patients presenting with worsened conditions and at more advanced cancer stages, in which treatment options tend to be costlier. The ‘double hit’ faced by patients here do not only stem from financial burdens but also from the consequences of treatment delay or interruptions and the inevitable heightened risk of COVID-19 infection; accompanied by the additional complications arising as a result of the infection [7].

Similar to countries around the world, the various forms of movement restrictions that were enforced have aggravated the rates of unemployment, loss of wages and the limited access to support from family or friends around Southeast Asia. Notably, unemployment rates have spiked to almost unprecedented levels; compared to 2% and 0.6%, respectively, before the pandemic in Laos and Thailand, unemployment rates were recently estimated to be around 25% in both countries [28]; in Vietnam, unemployment rates were at a 10-year high, with an estimated 5 million jobs lost in just the first quarter of 2020 [23]; in the Philippines, 7.3 million Filipinos had lost their job due to the pandemic, with a record-high unemployment rate of 18% [29]; while in Malaysia, which recently recorded the highest unemployment rate since 1989, an estimated 830,000 people are currently unemployed [30]. In Indonesia, the average household income fell by an estimated 24%, pushing an additional 35.9 million population into poverty [31]; while in Singapore, lower wealth households experienced a 10% reduction in income [32]. The economic impact of COVID19 hits even harder on the large proportion of the population in the region who work in the informal sector, comprising daily wage workers, temporary contract workers or small family-run businesses [28]. These vulnerable households are often one paycheque or one episode of illness away from financial catastrophe. It is therefore conceivable that the pandemic will have pushed more cancer patients and their families who were previously financially solvent, albeit precariously, into economic ruin.

Many cancer patients also face numerous issues while working following a diagnosis with cancer. We recently found that among Malaysian breast cancer survivors, besides experiencing catastrophic loss of household income, many recounted facing discriminations at the workplace due to their cancer [19], such as being forced to resign. It is not unthinkable that the pandemic may only have worsened the existing workplace discrimination when employers are forced to weigh in the organizational goals against the individual sufferings of employees with cancer. There have indeed been documentations of cancer survivors experiencing long-term cancer-related job loss, induced by a less accommodative work atmosphere, and reduced efficiency at work, commonly experienced by cancer survivors compared to patients without a history of cancer [7].

In light of the current COVID-19 pandemic that had brought about rising unemployment and catastrophic economic strain, the lack of a robust social security system in many Southeast Asian countries, especially in terms of income protection, could ultimately force many cancer survivors to choose between paying for their treatments, or to forego treatments, and feed their families. Discrimination and stigma surrounding cancer can further drive people with cancer to hide their cancer diagnosis from their employers or avoid seeking treatments at the hospital in order to retain their jobs. The delay and postponement in receiving a cancer diagnosis, active treatments and routine follow-ups, coupled with the rising unemployment and economic strain on household income, may lead many cancer patients in this moderately religious and culturally rich region to turn to traditional, alternative and/or complementary medicines. Use of traditional, alternative and/or complementary medicines among cancer patients in Southeast Asia has been reported to be as high as 85% [33]. Besides having important implications on health outcomes [34, 35], this is particularly alarming in light of country-specific findings from the ACTION study in which expenditures on traditional and complementary medicine doubled the incidence of financial catastrophe among Malaysian patients with cancer who sought care in the public hospitals [36].

## Potential solutions

Early identification of cancer survivors who may be financially vulnerable from the double financial hit due to the financial toxicity associated with cancer and treatments as well as the economic strains from the COVID-19 pandemic is crucial. One potential solution will be the use of the Comprehensive Score for Financial Toxicity (COST) questionnaire to determine the level of financial toxicity in patients. This is an 11-item measure of financial toxicity with items encompassing financial expenditure, financial resources and psychological response of cancer patients [37].

There needs to be a clear measurement of financial toxicity within affected families, with proper documentation of the socioeconomic influences on these patients and their families. This questionnaire can be a screening instrument to identify patients at a greater risk of financial toxicity, who can then be adequately referred to the appropriate support programs to assist in managing the financial strains of these patients. COST measures that indicate increased financial strains documented patients using coping strategies to manage the inflated medical costs incurred. Understanding the levels of financial toxicity through the COST questionnaire can aid in contributing to policy developments and through that at least reduce the impact in this population [38, 39].

Introduction of financial navigation programs may also be helpful to combat the financial impact of cancer and COVID-19. Although some form of financial counselling or assistance programs for cancer patients may be available in certain settings in Southeast Asia, they are usually focused on a specific episode of cancer care, rather than on the patient’s overall treatment plan [40]. Furthermore, many of these programs are also usually limited to a specific population, such as the uninsured or those with severe disease status, leaving out those who are underinsured or with early-stage cancer, who are also vulnerable to financial toxicity.

Financial navigation programs differ from the traditional and more limited financial counselling programs by proactively reaching out and developing comprehensive plans to meet each patient’s unique financial needs [40]. Several case studies on financial navigation programs have been reported and published [39-41]. For example, a financial navigation program was implemented through the Patient Advocate Foundation in the United States, in which a financial navigator provided support in areas such as monetary management, disability, employment, insurance coverage and psychosocial assistance. Through the financial navigation program, patients have reported savings of up to $12,000, along with nonmonetary benefits, such as improved emotional well-being [40]. Patient satisfaction on the program and its modules was also reported to be high [42]. Importantly, financial navigation programs do benefit not only the patients but also the institutions that offer the services, which have reported noteworthy savings of up to $4 million per annum; eventually covering the cost incurred in running the program [40].

It is strongly felt that financial navigation programs for people living with cancer in Southeast Asia should have a focus on increasing financial literacy, assessing risk of financial toxicity, optimizing health insurance, connecting patients to available financial resources and helping with needed paperwork to apply for financial assistance.

## Conclusion

Early identification of cancer patients experiencing financial toxicity following the pandemic will enable timely and appropriate interventions to be undertaken by various stakeholders, potentially averting a cascade of other economic fallouts that may last for years after cancer treatment. Detailed examination of health insurance status and employment-related factors contributing to financial toxicity in the low- and middle-income countries such as in Southeast Asian settings may allow tailoring of interventions based on individual patient attributes and economic background instead of adopting a ‘one size fits all’ approach that may not be very efficient.

## Conflicts of interest

The authors declare that there is no conflict of interest regarding the publication of this article.

## Funding statement

No funding was received for this article.
